# Physical disability and psychosocial impact due to chronic filarial lymphoedema in Sri Lanka

**DOI:** 10.1186/1475-2883-6-4

**Published:** 2007-03-29

**Authors:** RS Wijesinghe, AR Wickremasinghe, Sriyani Ekanayake, MSA Perera

**Affiliations:** 1 Department of Parasitology, Faculty of Medical Sciences, University of Sri Jayewardenepura, Gangodawila, Nugegoda, Sri Lanka; 2 Department of Community Medicine, Faculty of Medicine, University of Kelaniya, P.O. Box 6, Thalagolla Road, Ragama, Sri Lanka; 3 Department of Family Medicine, Faculty of Medical Sciences, University of Sri Jayewardenepura, Gangodawila, Nugegoda, Sri Lanka

## Abstract

**Background:**

Information on the physical and psychosocial disability of lymphatic filariasis in Sri Lanka is scarce. Therefore this study was carried out to describe the physical disability and psychosocial impact associated with chronic lymphoedema in patients attending filariasis clinics in the Colombo district, Sri Lanka.

**Methods:**

Four hundred and thirteen patients with lymphoedema of limbs attending filariasis clinics in Werahera and Dehiwala in the Colombo district were enrolled in the study after obtaining informed written consent. Data were collected using a pre-tested, interviewer-administered questionnaire and analyzed using SPSS.

**Results:**

Majority (95%) of patients had lower limbs affected and there was a significant association with difficulty in walking (p = 0.023). The swollen limb affected the work of 87 (52 %) of employed patients and 26 persons reported loss of job. Approximately 25 % and 6 % reported having problems interacting with the community and family, respectively and 8.7 % felt that they were rejected by society. The swollen limb was perceived as a major problem by 36.8 % of patients. Of the married persons, 5.7 % and 6.2 % reported sexual and marital problems respectively, due to their swollen limb/s. Of those who had marital problems, 77.3% reported sexual problems as well (p < 0.001).

**Conclusion:**

Lymphoedema significantly affects physical, psychological and social functioning in affected individuals. Morbidity control, in addition to control of physical disability, should target the psychosocial consequences.

## Background

Lymphatic filariasis (LF) is an important public health and socio-economic problem worldwide. It affects 120 million people in over 80 countries [1], of which, about 14 million suffer from lymphoedema or elephantiasis of legs [2]. The disease is prevalent in urban and rural areas affecting people of all ages and both sexes, particularly those of low socioeconomic status [3].

Although LF does not cause immediate mortality, the associated severe morbidity has resulted in it being recognized as the second leading cause of disability worldwide [4]. The two most common chronic manifestations of the disease – hydrocoele and lymphoedema cause socio-psychological problems to patients and their families [5]. Chronic disease is debilitating, leading to a restriction in the duration and capacity to work and to changes in activity patterns [6,7].

As morbidity management of individuals already affected by LF is an important component of the Global Programme for Elimination of Lymphatic Filariasis [8], it is important to understand the extent of the physical, psychological and social disability caused by the disease. Studies done in India and Ghana have demonstrated the functional impairment, disability [9], economic burden [10-12] and psychosocial burden [13,14] caused by chronic filarial lymphoedema on affected individuals, their families and communities.

In Sri Lanka, more than half the population, approximately 9.8 million people, live in the filariasis endemic area, which covers eight of the 25 districts of the country (T.S. Liyanage, unpublished observation). Information on the physical and psychosocial disability of filariasis in Sri Lanka is scarce. A study done in the south of Sri Lanka [15] revealed some of the socio – psychological problems experienced by lymphoedema patients. In this study, we have attempted to describe and quantify the physical and psychosocial disability caused by chronic filarial lymphoedema in the Colombo district in Sri Lanka.

## Methods

### Study area

The study was carried out in two of the three filariasis clinics conducted in the Colombo district by the national Anti-Filariasis Campaign. The commercial capital of Sri Lanka, Colombo, is located in this district, which is an area endemic for bancroftian filariasis. These filariasis clinics provide services to the people for screening and management of filariasis for both first contact care and referrals.

### Study design

A cross sectional descriptive study was carried out between April 2004 and February 2005 in the filariasis clinics at Werahera and Dehiwala in the Colombo district. Most of the clinic attendees of these two clinics had been referred by General Practioners (GPs), consultants or government hospitals after treating them for varying periods ranging from months to years with repeated courses of diethylcarbamazine citrate (DEC). Some patients had come direct for first contact care while others were self-referrals following treatment failures.

Lymphoedema was diagnosed clinically as persistent oedema of more than 6 weeks duration. Four hundred and thirteen patients were enrolled after obtaining informed written consent. Inclusion criteria were, patients should be above 5 years of age, attending the above filariasis clinics and have clinically detectable, persistent oedema of more than 6 weeks duration. Exclusion criteria included patients with bilateral leg involvement in whom other causes of oedema (eg: cardiac, renal, hepatic causes) had not been excluded and patients unable to understand/answer questions asked in questionnaire e.g.: mentally unstable patients. All patients selected were enrolled in the study except two patients with Down's Syndrome who were excluded from the study. In all patients with bilateral leg involvement other causes of oedema had already been excluded.

### Study instrument

As standard questionnaires to measure psychological impact of chronic lymphoedema have not been validated for Sri Lanka, a questionnaire was developed by a group of experts including a psychologist, a psychiatrist, a sociologist and a public health specialist. Face validity was assessed by an independent group who considered the questionnaire to be able to detect psychological impact. The questionnaire was developed to obtain information on demographic characteristics, disabilities (activities of daily living and impact on occupation, education, attending social gatherings, religious activities, leisure activities), social implications (interactions with family, friends, work mates, community), feelings regarding illness and marital and sexual problems. The questionnaire was pre-tested. The principal investigator and two trained medical officers administered the questionnaire.

The level of lymphoedema was graded according to standard criteria [16]:

Grade 1 – oedema spontaneously reversible on elevation

Grade II – oedema not spontaneously reversible on elevation, skin not thickened

Grade III – oedema not spontaneously reversible on elevation, skin thickened

Grade IV – oedema not spontaneously reversible on elevation, skin thickened with warty/nodular, papillomatous growths.

### Data analysis

Data analysis was done using SPSS version 10.0 of the SPSS software package (SPSS Inc, Chicago, IL). Frequency distributions were obtained and associations tested using the chi square test and t-tests.

### Ethical considerations

Ethical clearance for the study was obtained from the Ethical Review Committee of the Faculty of Medical Sciences, University of Sri Jayewardenepura, Sri Lanka.

All patients were treated for filariasis and associated problems and necessary referrals done when indicated. They were educated regarding the morbidity control measures advocated in the Community Home Based Care programme [2] and were followed up one year later.

## Results

### Profile of patients

The majority of patients were female (72.4%) and married (85.5%) (Table 1). The mean age of the patients was 51.27 years (SD = 13.58), ranging from 9 to 85 years. The majority of patients (n = 357, 86.4%) with lymphoedema were over 35 years of age (Table 2). Most females (64.2%) were housewives, while the majority of males (77.2%) were employed. Overall, 40.4% (n = 167) of the study sample was employed in paid jobs. Of those who were willing to reveal their income (n = 159), almost 40% were earning a monthly income of less than Rs. 5000 (≅ USD 50). The majority (65.6%) had studied up to grades 6–11.

**Table 1:** Profile of patients

Variable	Number (n = 413)	Percent
Age (years)		
≤ 35	56	13.6
35.1–50	131	31.7
50.1–65	168	40.7
> 65	58	14.0
Sex		
Male	114	27.6
Female	299	72.4
Marital status		
Married	353	85.5
Single	60	14.5
Educational level		
No schooling	16	3.9
Grades 1–5	68	16.5
Grades 6–11	271	65.6
A' level	43	10.4
Tertiary education	15	3.6
Monthly Income (Rs)^α^		
< 5,000	63	39.6
5,001 – 10,000	56	35.2
10,001 – 20,000	29	18.2
> 20,001	11	6.9
Occupation		
Professional, technical and related workers	14	3.4
Administrative and Managerial workers	3	0.7
Clerical and related workers	7	1.7
Sales workers	55	13.3
Service workers	30	7.3
Agricultural, animal husbandry, forestry, fishermen and hunters	6	1.5
Production, transport equipment operators and labourers	48	11.6
Armed forces personnel	4	0.9
Workers not reporting any occupation	49	11.9
Students	5	1.2
Housewives	192	46.5
Site of lymphoedema^β^		
Right lower limb	210	50.8
Left lower limb	245	59.3
Right upper limb	20	4.8
Left upper limb	17	4.1
Lymphoedema Grade^β^		
Grade I	170	41.2
Grade II	205	49.6
Grade III	92	22.3
Grade IV	25	6.1
Duration of lymphoedema (years)		
≤ 1	42	10.2
1–5	115	27.8
≥ 5	256	62.0

^α ^There were 167 employed in paid jobs but only 159 of them reported their earned income.

^β ^81 patients had lymphoedema of more than one limb

**Table 2:** Distribution of lymphoedema in age groups

Age (years)	Number of patients with lymphoedema (n = 413)	Number (% within age group) of patients with lymphoedema of maximum grade			
		
		I	II	III	IV
5 – 15	03	02 (66.7)	01 (33.3)	-	-
15.1 – 25	09	06 (66.7)	03 (33.3)	-	-
25.1 – 35	44	24 (54.5)	17 (38.6)	02 (4.5)	01 (2.3)
> 35.1	357	233 (65.3)	61 (17.1)	43 (12.0)	20 (5.6)

### Characteristics of lymphoedema

The duration of lymphoedema in a limb varied from 5.27 to 11.64 years with the longest duration being 50 years. The left (59.3 %) and right (50.8%) lower limbs were the most frequently affected (Table 1). Eighty one patients had lymphoedema of more than one limb. The majority of the limbs with lymphoedema (49.6 %) were of Grade II and Grade 1 (41.2%).

### Impact on activities of daily living

Approximately 37% reported difficulty in walking (Table 3) and this was significantly associated with lower limb oedema (p = 0.023) as compared to patients without lower limb oedema. Almost 30 % reported difficulty in doing housework due to the swollen limbs, but this was not significantly associated with lower limb or upper limb oedema. The maximum grade of lymphoedema was significantly associated with difficulty in standing (p = 0.008) and using the toilet (p = 0.005). Although not significant, more patients with grades III and IV lymphoedema found it difficult to bathe than patients with lower grades of lymphoedema.

**Table 3:** Impact of lymphoedema on activities of daily living

Activity of daily living	Number (%) of persons who		
	
	Have no difficulty (n = 413)	Can do with difficulty (n = 413)	Cannot do without help (n = 413)
Eating	413 (100)	0	0
Dressing	406 (98.3)	7 (1.7)	0
Bathing	397 (96.1)	16 (3.9)	0
Using toilet	341 (82.6)	70 (16.9)	2 (0.5)
Walking	262 (63.4)	151 (36.6)	0
Doing house work	291 (70.5)	122 (29.5)	0
Standing	387 (93.7)	26 (6.3)	0

The other activities that patients reported difficulty in performing were, climbing stairs, walking uphill, drawing water from well, getting into a bus, holding railing in a bus, sitting and lifting heavy objects

### Interference with selected activities

All students interviewed in this study reported that the lymphoedema interfered with their education (Table 4). This was mainly due to the patients feeling that their fellow students rejected them (less friendly/isolated/no spontaneous invitation to join in group activities), resulting in them not following classes regularly. An affected medical student reported difficulty in standing for prolonged periods during ward work.

**Table 4:** Psychosocial impact of lymphoedema

Impact	Number	Percent
**Interference with selected activities**		
Occupation (n = 167)	87	52.1
Education (n = 5)	5	100.0
Religious activities (n = 413)	65	15.7
Leisure activities (n = 413)	30	7.2
Attending social gatherings (n = 413)	77	18.6
**Difficulties in interacting with categories of society**		
Family (n = 413)	24	5.8
Friends (n = 413)	30	7.3
Work mates (n = 167)	34	20.4
Community (n = 413)	104	25.2
**Patients' feelings regarding the swollen limb^α β^**		
Feeling shy	82	33.3
Severely restricting daily activities	70	28.5
Social stigma	71	28.9
Perceived as major problem affecting their lives	152	61.8
Depressed	21	8.5
Fear of elephantiasis	18	7.3

^α ^an individual could have specified more than 1 feeling

^β ^Percent calculated among the 246 patients who stated that the swollen limb was a problem to them

Of the 167 employed patients, more than half (n = 87, 52.1%) reported interference with their occupation due to the swollen limb/s. This was in the form of loss of work time, inability to do heavy work, stigmatization, being penalized by supervisors and co-workers and being given less overtime work. Twenty six patients, comprising 12.3% of those currently employed, unemployed and retired, claimed they lost their jobs due to the lymphoedema. Most of those who had lost jobs were labourers doing heavy work, but also included a photographer and an artist who had to give up their professions due to upper limb swelling and an army personal who had to give up active combat due to lower limb swelling. The majority of those who lost their jobs were married (69.2%), males (61.5%) with almost 20 % (of those who stated an income) previously having earned a monthly income of less than SL Rs. 5000 (≅ USD 50).

The lymphoedema also interfered with religious activities in 16 % of affected patients with more impairment in higher grades of lymphoedema (p = 0.005). This was due to inability to squat to worship in Buddhist temples, as well as being reluctant to go to places of worship for fear of others noticing their swollen limb.

Leisure activities such as sports and other recreational activities were also affected due to the physical difficulty in doing such activity as well as feeling shy to interact with peers. Although not significant, a greater percentage of patients with grade IV lymphoedema had interference with leisure activities and occupation than patients with lower grades of lymphoedema.

### Interactions with society

Twenty five percent of the patients found it difficult to get along with the community (Table 4) and 36 (8.7%) felt they were totally rejected by society (laughed at, not included in social activities, isolated). Even within their own families, 24 (5.8%) patients had problems interacting with them.

### Patients' feelings regarding the swollen limbs

Almost 60 % (n = 246) of the patients perceived their swollen limbs as being a problem affecting their lives and had varying feelings regarding it (Table 4). Twenty one (8.5%) patients reported feelings of depression, of whom five had past suicidal thoughts. The other feelings/concerns expressed were inconvenience in always having to wear long garments to hide the swollen lower limbs, inability to wear normal foot wear, feeling of being a burden to the family, fear of transmitting disease to family, inability to do a job, intense fear of being rejected by society, worried about not being able to get married due to the swollen limb and that their whole life changed due to the illness. Fifty two (12.6%) patients (12% of females and 14 % of males) reported feeling lonely and isolated because of the swollen limb.

### Marital and sexual problems

Of the 353 married patients, 20 (5.7%) and 22 (6.2%) reported sexual and marital problems respectively, which they attributed to their swollen limbs. Sexual problems were inquired only from married individuals due to cultural reasons. More females than males reported these problems (female: male ratio of sexual problems was 12:8 and marital problems was 17: 5) although this was not statistically significant (p = 0.333 and p = 0.562 respectively). Among married persons, sexual problems were significantly associated with marital problems, with 77% of those who reported marital problems also having sexual problems (p < 0.001).

The marital problems varied from the spouse nagging and being unsupportive towards treatment to totally rejecting and avoiding all physical contact with the patient. Three patients reported that their husbands had left them for other women due to this problem.

## Discussion

Lymphatic filariasis causes disability due to its acute manifestations as well as its chronic forms. Unlike the physical disabilities that are visible, the psychosocial disabilities caused by LF tend to be unrecognized [14]. There is growing interest in the role of mental and social health in promoting positive health and good quality of life [14]. Previous studies have shown that LF causes severe disability and considerable treatment costs due to acute and chronic disease in India [5,9,10,13,17,18] and Ghana [12]. Such information is scarce in Sri Lanka.

It has been shown that medical experts assigned lower severity levels to patients' physical and psychosocial disability than the sufferers, indicating that experts are able to perceive the extent of disability to a certain extent only [14]. As only patients can assess the exact extent of disability, their perceptions are important in planning and implementing morbidity control programmes for LF [14]. This study demonstrates the significant physical disability and psychosocial problems as perceived by patients affected by chronic filarial lymphoedema in an endemic urban area of Sri Lanka.

Difficulties were reported in all activities inquired about, mostly in walking which was significantly associated with lower limb oedema and also in carrying out household chores. Similar results have been reported from rural India, where domestic activities of affected females were seen to be impaired due to chronic and/or acute manifestations of the disease [5,7,9,17,18]. Many patients in our study population were using toilets of the squatting type, which posed problems for many of them, with two patients not being able to use the toilet at all without help from others.

These physical difficulties arose in spite of the majority of patients interviewed suffering from a relatively early stage of lymphoedema of Grade II, which shows that even at lower grades, lymphoedema causes physical disability. The higher the grades of lymphoedema, the difficulty in doing activities such as standing, using the toilet and bathing were more. Therefore, it is important to manage the lymphoedema at very early stages, preferably at grade I, to prevent progression and increase of disability.

Lymphoedema affected the occupation in more than half the patients in our study. Twenty six patients had completely lost or left their jobs, the majority of them being married males, the sole breadwinners of the family, who worked as labourers doing heavy work and earning less than SL Rs. 5000 (USD 50) a month. Therefore losing that small source of income would have clearly had a very significant social impact on the affected patients' families. A greater percentage of patients with grade IV lymphoedema had interference with their occupational activities than patients with lower grades of lymphoedema, which is to be expected. In India too, several studies have reported similar occupational interferences and loss of jobs due to the chronic lymphoedema as well as recurrent acute adenolymphangitis attacks (ADL attacks) [7,9,13,14,17]. Loss of work time in chronic patients, unlike in the acute manifestations, is perpetual as the chronic disease manifestations are mostly irreversible [5].

The impact on social activities such as attending social gatherings and leisure activities as seen in our study were more in higher grades of lymphoedema. This trend is to be expected as the more severe the lymphoedema is, the more the accompanying complications and disability.

Almost one fourth of patients had problems interacting with the community. This resulted in patients, especially those with advanced stages of lymphoedema/elephantiasis, becoming isolated and withdrawn from society, avoiding social gatherings and activities and becoming angry, bitter and depressed about their situation. Even within their own families, about 6 % reported problems arising due to their swollen limbs.

Similar results were reported from the Gampaha district of Sri Lanka [19], where 18.2% of patients experienced feelings of being rejected by society. In India too, chronic brugian filariasis prevented people from gaining a standing in the community including claims that they did not receive any support from their community [13].

Approximately 37% of patients in this study felt the swollen limb was a major problem affecting their lives, causing shyness, depression and intense worry about progression of disease to elephantiasis. A few had been suicidal as well at some point of their disease. Similar negative feelings have been reported in studies done in other areas of Sri Lanka [15,19], India [13,14] and Ghana [20].

Approximately six percent of married persons experienced sexual and marital problems with the cause of the marital problems probably being sexual problems arising due to the swollen limb/s (p < 0.001). The patients view was that these problems were either solely or mainly due to their swollen limb/s. It was seen that more females than males reported marital and sexual problems in our study. This was not surprising as males are generally less reluctant to admit and discuss their problems, while females are more responsive towards admitting these issues and discussing them. Similar problems such as unsuitability for marriage, sexual dysfunction and divorce attributed to the lymphoedema have been reported in a study in Ghana [20].

Apart from the chronic effects, acute ADL attacks too cause temporary but severe disability in patients [14]. Eighty three percent of acute episodes occur in chronic patients [21]. We specifically do not address these issues in this manuscript. Therefore, if acute manifestations were also considered, the disability and impact of chronic lymphoedema would be much greater than what is highlighted here.

Due to the fact that the disabling psycho-social impact of chronic lymphoedema often go unnoticed, more emphasis is given to treating the patient's visible physical problems, often resulting in irrational continuous prescription of anti filariasis drugs. This was clearly seen in our study population, the majority of whom were treated with DEC for very long periods of time, the maximum being 43 years.

The study was conducted in the Colombo district, the district having the best educational and health facilities and over 90% of the population being literate. In spite of this educational background, many patients are stigmatized and penalized even in relatively enlightened communities.

The WHO recommended morbidity control strategy in the form of a Community Home Based Care (CHBC) programme is still in its infancy in Sri Lanka. The CHBC programme deals with alleviating the physical disability of already affected patients by motivating and training patients and their carers to adhere to simple, yet effective strategies involving the affected areas – washing, elevation, prevention and treating entry lesions and using foot wear [2]. While fully appreciating the importance of this and further reiterating the importance of focusing on new strategies in the management of lymphoedema to alleviate physical morbidity, we strongly recommend that the psychosocial problems of patients be addressed at the same time, adopting a holistic approach. Counselling, formation of support groups and rehabilitation of affected patients should be done. Finding them less strenuous, preferably home based jobs would reduce patients' economic problems and give them self-confidence and a feeling of independence and worth. The community should be educated on the cause and transmission of the disease to dispel misconceptions, myths and stigma attached to the disease. The patients too should be educated to adjust to the effects of the illness and treatment.

We were unable to examine patients for genital manifestations in the clinic setting, which did not provide adequate privacy to patients. We acknowledge this to be a major limitation of this study as genital manifestations causes sexual disability in affected patients. [22].

These results would be a useful guide to design a morbidity control programme suited to the Sri Lankan community. Family members should be trained and involved in these morbidity management programmes, especially the CHBC, to reduce the physical disability directly and accompanying psychosocial disability indirectly.

## Conclusion

A significant proportion of patients with chronic filarial lymphoedema in the Colombo district in Sri Lanka suffer some form of physical disability, psychological and/or social consequences due to the illness, despite having been treated conventionally with long term DEC. The management of chronic lymphoedema should include measures to alleviate the psychosocial problems as well as the physical morbidity of affected patients.

## Abbreviations

ADL: Adenolymphangitis

LF: Lymphatic filariasis

GP: General Practioners

DEC: diethylcarbamazine citrate

SL Rs: Sri Lankan rupees

USD: US Dollar

CHBC: Community Home Based Care

## Competing interests

The author(s) declare that they have no competing interests.

## Authors' contributions

**RW **conceived and designed the study, collected data, analyzed and interpreted data and drafted the manuscript.

**ARW **analyzed and interpreted data, supervised drafting of manuscript and revised manuscript critically for intellectual content.

**SE **conceived, designed and supervised the study and revised manuscript critically for intellectual content.

**MSAP **supervised the study and revised manuscript critically for intellectual content.

All authors read and approved the final manuscript.
